# Living Arrangements and Gendered Work Prospects Among Chinese Grandparents

**DOI:** 10.1093/geroni/igab046.1901

**Published:** 2021-12-17

**Authors:** Jing Ye, Feinian Chen

**Affiliations:** 1 University of Maryland College Park, University Park, Maryland, United States; 2 University of Maryland College Park, University of Maryland, Maryland, United States

## Abstract

Recent literature on grandparenthood in China overwhelmingly focuses on the role of grandparents as caregivers for grandchildren. However, many become grandparents at an age when they are still active in the labor force. Using data from the China Health and Retirement Longitudinal Study (2011-2015), this study examines the extent to which coresidence with grandchildren affects grandparents’ labor force participation and work hours. Results from our fixed-effect models show that, living with grandchildren has a positive effect on men’s work participation and hours worked, especially for those with flexible jobs. For women with inflexible jobs, coresidence with grandchildren has a negative impact on their work prospect. Furthermore, grandparents in skipped generational households are less likely to scale back in work than those in multigenerational households, indicating a high level of double burden from both work and caregiving responsibilities. Our study extends prior work by emphasizing grandparents’ role as active workers and highlights the importance to understand work and caregiving demands in a gendered and dynamic household context.

